# A Comparative Study between Polymer and Metal Additive Manufacturing Approaches in Investigating Stiffened Hexagonal Cells

**DOI:** 10.3390/ma14040883

**Published:** 2021-02-12

**Authors:** Othman Laban, Elsadig Mahdi, Samahat Samim, John-John Cabibihan

**Affiliations:** 1 School of Mechanical and Manufacturing Engineering, the University of New South Wales, Sydney, NSW 2052, Australia; 2 Mechanical and Industrial Engineering Department, College of Engineering, Qatar University, Doha 2713, Qatar; elsadigms@qu.edu.qa (E.M.); samahat@qu.edu.qa (S.S.); john.cabibihan@qu.edu.qa (J.-J.C.)

**Keywords:** 3D printing, additive manufacturing, crashworthiness, energy absorption, direct metal laser sintering, fused deposition modeling, effective method

## Abstract

Recent polymer and metal additive manufacturing technologies were proven capable of building complex structures with high accuracy. Although their final products differ significantly in terms of mechanical properties and building cost, many structural optimization studies were performed with either one without systematic justification. Therefore, this study investigated whether the Direct Metal Laser Sintering (DMLS) and Fused Deposition Modelling (FDM) methodologies can provide similar conclusions when performing geometrical manipulations for optimizing structural crashworthiness. Two identical sets of four shapes of stiffened hexagonal cells were built and crushed under quasi-static loading. The results were compared in terms of collapsing behavior, load-carrying performance, and energy-absorption capability. Although the observed failure modes were different since the base-materials differ, similar improvement trends in performance were observed between both fabrication approaches. Therefore, FDM was recommended as a fabrication method to optimize thin-walled cellular hexagonal parameters since it was 80% more time-efficient and 53.6% cheaper than the DMLS technique.

## 1. Introduction

Additive Manufacturing (AM) has become a popular rapid prototyping technology that can produce lightweight complex geometries [1]. This, in turn, leads to almost non-restricted abilities to improve the mechanical properties of cellular structures with materials varying from different types of polymers to metals [2]. Fused Deposition Modelling (FDM) and direct metal laser sintering (DMLS) are the cutting edge technologies used for printing polymeric and metallic structures, respectively. They have been extensively applied in the field of biomaterials, aerospace, and automotive industries [3,4].

The FDM technology consists of depositing molten polymer material on a platform in a layer-by-layer fashion to create the object [5]. On the other hand, the DMLS technology creates an object in a similar layup but through the fashion of metallic nano-powder on a printing bed with a high-power laser beam [1,6]. While both technologies offer manufacturing flexibility, FDM is characterized by its affordable costs as compared to the DMLS which has a relatively higher energy consumption and material cost in addition to post processes like heat treatments [6,7].

Although the FDM and DMLS technologies are diverse in their technique and material properties, the recent literature indicates both are being utilized for similar research purposes. For instance, the former AM process was used in investigating the compressive strength and energy absorption of square and hexagonal polymer structures by varying the cell edge thickness [8]. A similar research trend was conducted with the DMLS for investigating the energy absorption capabilities of aluminum alloy lattice structures by varying their geometrical parameters [9,10]. Another example was seen in an optimization study of polymeric polyurethane cellular structures for personal protective equipment application, where their collapsing behavior was analyzed with functionally graded AM models [11]. Interestingly, similar functionally graded geometries were optimized and manufactured by means of metallic AM techniques [12,13].

The previous comparison raises a concern about whether to choose the polymeric or metallic AM approach since the nature of experiments and study objectives in those investigations were similar. Indeed, the DMLS offers more capability to create non-prismatic and functionally graded elements or microstructures [1,14]. However, it is well known it is more energy consuming and requires post-preparation steps [15]. Nevertheless, the material handling and overall cost are also favoring polymeric AM techniques [16]. Therefore, the selection of the DMLS approach can be justified when functionally graded material properties are required through the structures. Otherwise, when the geometrical properties of the structure being investigated are the only varying parameters, it is not yet clear if it provides an advantage that leads to a distinguishing outcome over the FDM printing method.

This study aims to compare improvement trends of load-carrying capacity and energy absorption capability between the polymer and metal structural configurations. Two similar sets of thin-walled hexagonal configurations were built using FDM and DMLS AM technologies and then crushed under in-plane quasi-static load.

## 2. Experimental Methodology

**Figure 1:**
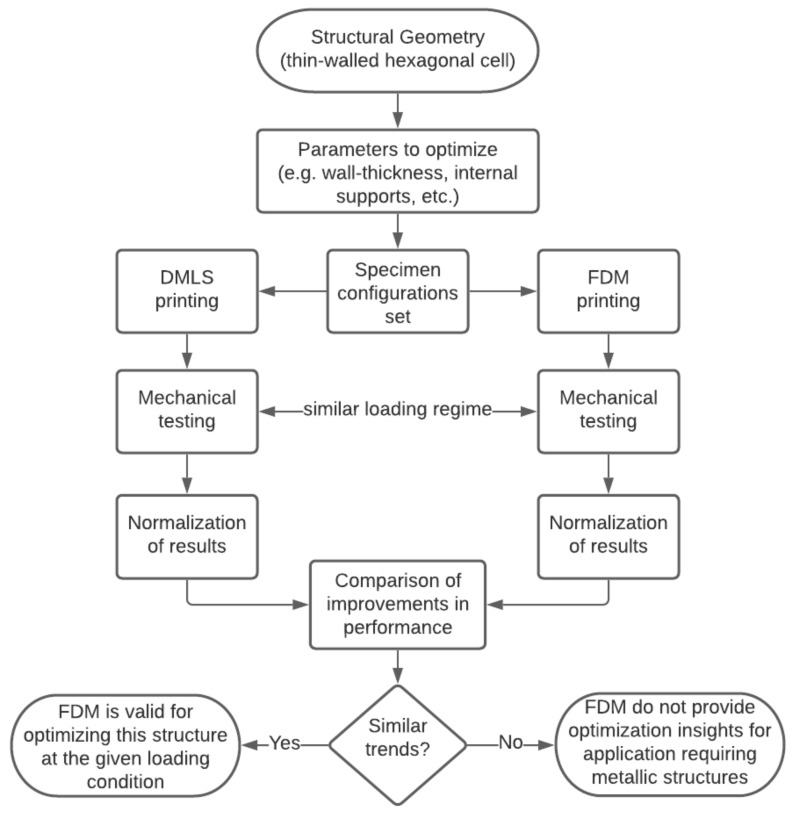
Decision tree for the proposed research investigation.

A flowchart of the methodology is presented in Figure 1, and more details are given in the subsequent subsections. Cellular hexagonal structures have been selected based on previous research work conducted in the crashworthiness field. The stiffening effect of increasing the number of internal supports on the structure performance was experimentally investigated using FDM and DMLS approaches to fabricate the specimens. The results were normalized prior to comparative analyses due to the vast difference between polymer and metal material properties. FDM approach can be suggested for providing optimizing insights into structural parameters of cellular hexagonal shapes if similar trends in crashworthiness performance with the DMLS approach were observed. Otherwise, the DMLS building technique must be taken if the application dictates the use of metallic materials (e.g., exposure to high temperatures).

### 2.1. Selected Geometric Configurations

**Figure 2:**
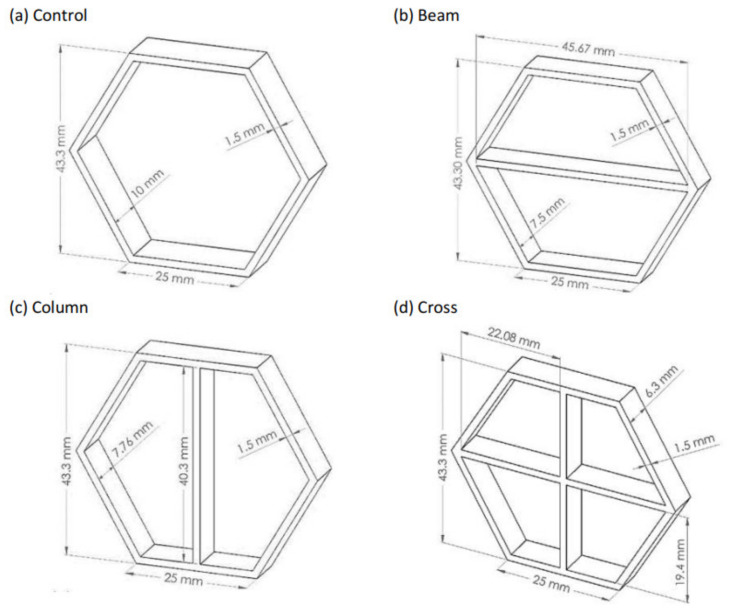
Geometrical configurations of the four hexagonal cells; (**a**) Control without stiffening, (**b**) cell with beam support, (**c**) cell with column support, and (**d**) cell with crossed support. The displayed dimensions are in mm.

Four geometric combinations of the honeycomb cells were studied for maximum Specific Energy Absorption (SEA) and other mechanical properties (Figure 2). The cellular height, width, and wall thickness were kept the same, while the depth for each configuration was adjusted to unify their weights. The coreless configuration (a) is the control structure, while (b), (c), and (d) are stiffened configurations having a beam, a column, and upright cross supports, respectively.

### 2.2. AM Technologies

**Figure 3:**
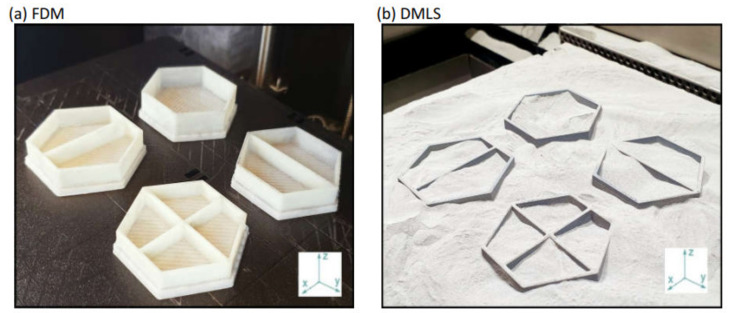
A photograph of the specimens on the building platform of (**a**) FDM and (**b**) DMLS machines. Upright (ZX) building orientation was chosen.

The polymeric specimens were printed by using FDM technology by Stratasys uPrint SE printer (Stratasys Ltd., Eden Prairie, MN, USA). The building material used by the printer is ABS plus P430 thermoplastic (Stratasys Ltd., Eden Prairie, MN, USA). The metal specimens were printed using DMLS technology by EOS GmbH EOSINT M280 printer (EOS GmbH, Munich, Germany), where the selected building material is AlSi10Mg Aluminum powder (EOS GmbH, Munich, Germany). The mechanical properties of both ABS and Aluminum materials are given in Table 1, while the machine parameters and chemical composition of AlSi10Mg powder are shown in Table 2. For DMLS samples, post-printing heat treatment was applied at 300 °C for two hours in the chamber as recommended by the EOS GmbH for relieving internal stresses and improve the ductility. Both printing techniques required the five key steps: developing a CAD model, converting it to an STL file, slicing and generating pathways, writing the g-code, and finally the printing process. A similar building direction, on the vertical z-axis, was selected as indicated in Figure 3. The geometry of the configurations did not require the need to use supports during the building process. This, in turn, has eliminated the need to use chemical agents for dissolving the support material for the FDM specimens or post-machining for the DMLS specimens.

### 2.3. Testing and Evaluation Procedures

The proposed configurations were subjected to in-plane compressive loading according to the ASTM E9-09 standard [17] using Instron 5585 (Instron Corporation, Norwood, MA, USA) universal testing machine. The specimens were placed between two 6-inch diameter flat platens. All configurations were compressed at a speed of 15 mm/min up to at least 90% of their height. Three tests of each aspect ratio and loading condition were carefully designed for data reproducibility, and the average result of the three tests was taken in each case. Load-displacement measurements were recorded every 1 ms by the Instron data acquisition system. Collapsing behavior of all configurations was captured via 60 frames per second camera.

Multiple performance parameters were adopted for mechanical performance assessment. The Initial Peak Force (IPF) parameter was used to indicate the amount of load required to initiate a permanent deformation in the structure [18]. In addition, great attention was directed to its instantaneous Crash Force Efficiency (CFE) [15]. This is expressed as the ratio of average force to the IPF, and it mainly reflects the stability of the structure in carrying the load throughout the compression stroke. Lastly, the SEA is the ratio of the total energy absorbed by the structure to the mass of the material. Since tested sample configurations were different from each other in dimensions and material, SEA was found to be a more suitable parameter comparing their energy absorption performance [19]. The total energy absorbed is obtained by integrating the force-displacement curve until the initiation of the densification phase.

## 3. Results and Discussion

### 3.1. Comparative Analysis of Collapsing Behavior

**Figure 4:**
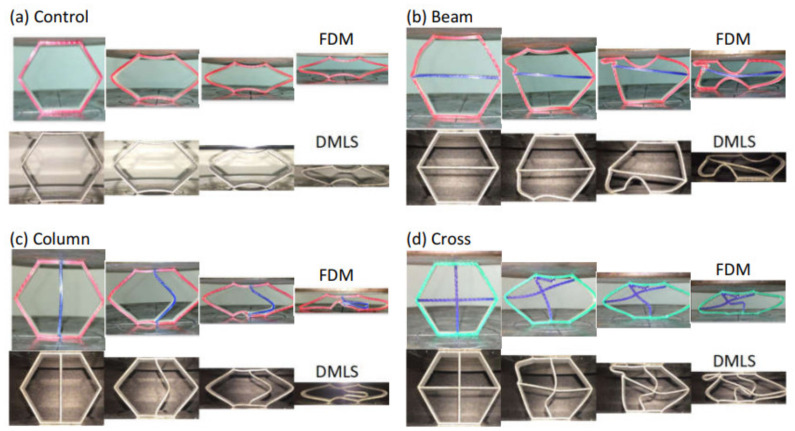
Crushing behavior of the two sets of honeycomb configurations under quasi-static axial compression. The first raw with the colored specimens are the polymer set while the bottom structures with the black background are the metallic set. (**a**) Control; (**b**) Beam; (**c**) Column; (**d**) Cross.

**Figure 5:**
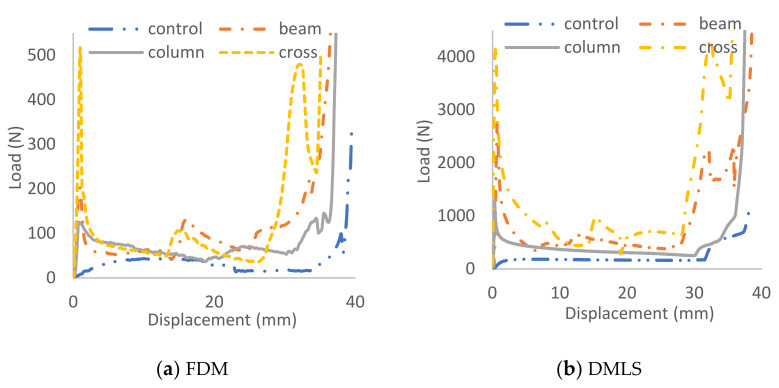
Compressive load-displacement history of hexagonal cellular configurations made with (**a**) FDM and (**b**) DMLS specimens.

The introduction of core supports has significantly altered the collapsing mechanism of the structure (Figure 4). The global outward expansion has occurred in the control coreless specimen, while localized buckling and material breakage were the dominant failure modes in the others. In general, nearly similar crushing behaviors were observed between DMLS and FDM specimens. Their load carrying trends were also relatively close, but with considerably higher magnitudes for the DMLS specimens due to their mechanical strength (Figure 5).

One discrepancy was noticed, that the FDM specimens have exhibited more material cracking owing to their low strain at failure compared to the DMLS (Figure 4). For instance, the control specimens began expanding sideways immediately after applying the load (Figure 4a) and the load was supported gradually by both materials (Figure 5). However, the load rose again towards the end stage only in the DMLS curve. That was caused by the contact of the upper and bottom beam members that were initially flat with the compression platen. However, this was not featured in the FDM specimen since its sides had fractured whilst expanding. The fabrication technique was not the cause of this variation in the behavior as the ductile nature of aluminum material is well known, especially after the employment of the post-printing heat-treatment [20]. Its fracture strain percentage is almost double the ABS (Table 1) and thus surely had contributed to this variation.

**Figure 6:**
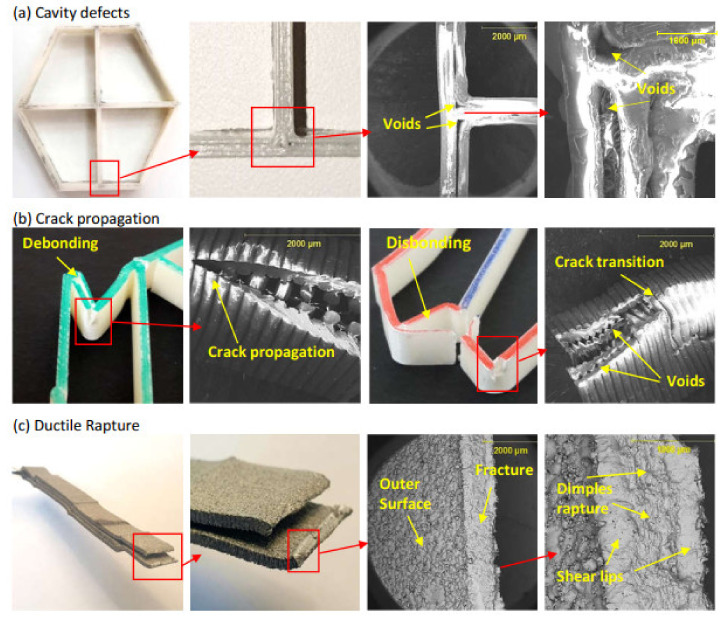
Scanning electron microscopy photographs highlighting (**a**) defects from FDM printing at the joints, (**b**) crack propagation perpendicular to printing paths and crack transition in-between those paths, and (**c**) material ductile rupture from excessive deflection in the DMLS specimens.

The fracture mechanics at the structural joints were inconsistent between the FDM and DMLS methods. The former specimens tend to break from the joints at an earlier stage. For instance, the right joint in the FDM cross configuration has a quick snap off at an early stage while the DMLS specimen joints have remained intact throughout the crushing stroke (Figure 4d). The quick breakage of the FDM joints indicates a presence of printing defects that weaken those joints. The FDM process generates multiple pathways for jetting the building material. The likelihood of unmapping the narrow areas by the fusing head is high in similar printing techniques, especially when building thin-walled structures [11]. Scanning electron microscopy (Thermo Fisher Scientific, Waltham, MA, USA) imaging has confirmed the presence of micro-voids in the cross-section of the joint which, in turn, are likely to promote higher stress concentrations (Figure 6a). In addition, the fusion between the wall thickness layers is not fully diffused since the injected material cools quickly before the next one is added, and thus causes poor adhesion with a higher risk of debonding to occur [21]. This was highlighted at the microscale in Figure 6b where the propagating crack has transitioned through delamination rather than material breakage. In addition, full layer debonding can be seen at the macroscale due to the presence of material discontinuities in-between the wall thickness layers (Figure 6b). In the DMLS technique, however, seamless diffusion at the microscale level was seen throughout the material cross-section without traces of voids at the joints were identified. This is most likely resulted by the improved layer thickness over the FDM and as a result of the stress-relieving heat treatment [22]. The dimples seen on the fractured surface in Figure 6c indicates a ductile rupture caused by the excessive extension of the outer surface where the tension was the greatest. The fracture has progressed diagonally at approximately 50° through the wall thickness. On the other hand, crack propagation with progressive brittle fracture was the dominant failure mode in the FDM specimens (Figure 6).

### 3.2. Performance of Configurations

**Figure 7:**
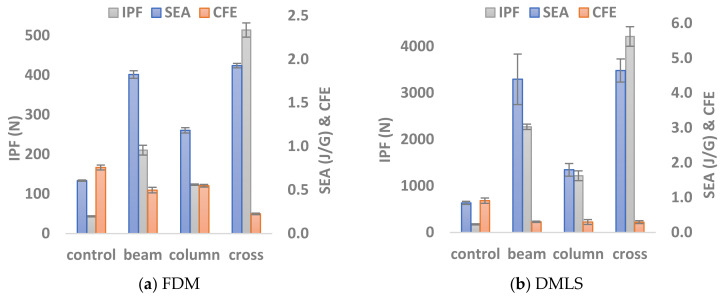
Bar charts summarizing the performance levels in terms of IPF, SEA, CFE exhibited by the (**a**) FDM and (**b**) DMLS specimens.

Significant variations were observed among the configurations in terms of their load-carrying capacity and energy-absorbing capability (Figure 7). Those variations were relatively higher for the specimen produced by the DMLS. For instance, introducing beam support to the coreless cell has restricted its sideways expansion and promoted localized buckling in its outer links (Figure 4b). For the FDM, this has improved the initial peak by 380% and the SEA by 199%, whereas the DMLS sample showed improvements of 594% and 418% for the same parameters, respectively. The column support, on the other hand, did not limit the sideways expansion; rather it started buckling as soon as the load was applied. This, in turn, gave a lower peak load followed by a smooth load decay as no fracturing occurred (Figure 5). Subsequently, it had a better CFE with a slightly lower peak load and SEA. When both were combined in the cross support (Figure 4d), it exhibited the highest peak load compared to the other configurations, almost double the value. This was followed by a rapid drop when the beam broke or the column buckled for the FDM and DMLS specimens, respectively. Then the structures were no longer able to carry high loads as indicated by their low average load index. This, in turn, has resulted in a low SEA and CFE.

**Figure 8:**
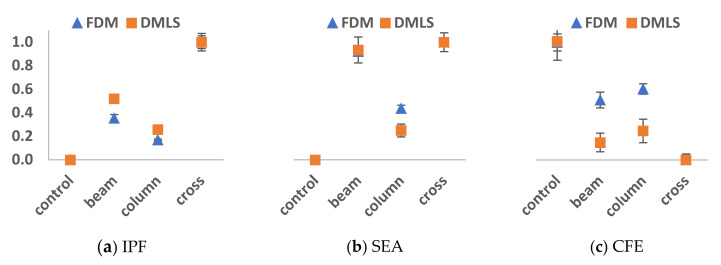
Comparative analysis between FDM and DMLS normalized performance indices: (**a**) IPF, (**b**) SEA, and (**c**) CFE.

Although the performance magnitudes of the selected cellular configurations were different for both printing techniques, their variations were relatively similar. Unity-based normalization technique (Equation (1)) was used to scale all the values to unity for more accurate comparison since the DMLS specimens tend to have higher performance values than FDM specimens. This has yielded similar conclusions. Looking at the trend of the normalized performance indices in Figure 8, the variations between the performances of the configuration is more vivid in the DMLS specimens. The overall trends are similar for both techniques except in the CFE for the beam and column configurations. This is because their peak load performance in the DMLS specimens was lower owing to the ductility of the central supports. The ranking of the peaks and valleys are similar which indicates that additive manufacturing techniques were able to highlight the optimal and worst configurations in terms of either one of the performance indices. Overall, similar conclusions were drawn from both additive manufacturing approaches.

(1) $$X_{normalized}=\frac{X_{i}-X_{\min\;}}{X_{max}-X_{min}}$$

### 3.3. Cost-Effectiveness

Printing periods for the twelve specimens were estimated to be six hours for the DMLS set and two hours for the FDM set obtained from the software (Materialise Magics [23] and CalatystEX [24]) that were used to generate the G-Codes. However, the actual printing time for the former was found to be higher since it required an additional one hour to prepare the machine and leveling the printing bed. Nevertheless, post-printing steps have consumed an additional three hours for releasing the samples from the bed and stress relieving in the heated oven. In total, the actual printing times were found to be 10 h for the DMLS configurations, whereas the FDM ones were printed in less than two hours. In other words, the latter printing technique was approximately 80% more time-efficient than the former.

Another essential factor is the cost associated with both techniques. The raw building materials were rated at 171 USD/kg for the AlSi10Mg powder (EOS) and 210 USD per canister (660 cm^3^) for the ABS P430 (Stratasys). The sum of the configuration’s material volume was 25.08 cm^3^ and their total weight was 100.62 g for the DMLS specimens. Hence, the printing cost roughly adds up to 7.98 USD for the FDM and 17.2 USD for the DMLS with an additional estimated 4.5 USD for the heat-treatment. This means the FDM printing was more than 53.6% cheaper than the DMLS technique, even without taking into account the additional costs for the required argon gas nor the technical personnel in-charge of pre- and post-printing steps, such as powder sieving and surfacing the building plate. Those were difficult parameters to measure and their rate depends on the country’s standards [6].

## 4. Conclusions

Two commonly used additive manufacturing techniques were selected to build two sets of specimens with similar configurations for energy absorption investigation. Comparative analysis between the two sets was performed in terms of the observed failure modes, IPF, CFE, and SEA. The following points were drawn:

- In general, the collapsing behavior of the configurations was similar between FDM and DMLS sets as well as the load-displacement trends.
- The failure modes were different where crack propagation with progressive brittle fracture was dominant in FDM specimens while the DMLS specimens had excessive deformation with the ductile fractures.
- Material discontinuity defects and poor diffusion between building layers were observed at the structural joints produced by the FDM technique whereas the DMLS built seamless transitioning between the layers.
- Overall, similar conclusions were drawn from the performance parameters of both sets. The cross configuration has the highest IPF and SEA with the lowest CFE in both FDM and DMLS cases. In contrast, the coreless hexagonal cell had the highest CFE while the IPF was compensated.
- The FDM printing was 80% more time-efficient and 53.6% cheaper than the DMLS technique.

In conclusion, the FDM is recommended for such geometrical optimization studies owing to the similarity in results observed in this study. Future work will investigate whether similar observations can be driven when both sets are compared with patterned structure configurations rather than at cellular levels. In lattice structures, individual cells interact with their neighboring cells and transfer loads, and thus the boundary conditions are different to great extent.

## Figures and Tables

**Table 1:** Mechanical properties of the ABS P430 and heat-treated AlSi10Mg measured parallel to the upright (ZX) building orientation, provided by Stratasys and EOS, respectively.

Mechanical Property	ABS (P430)	AlSi10Mg (HT)
Specific gravity	1.04	2.67
Ultimate tensile strength	33 MPa	350 MPa
Yield strength	31 MPa	230 MPa
Modulus of elasticity	2.2 GPa	60 GPa
Failure strain	6%	11%

**Table 2:** EOS M280 3D machine parameters and material composition of AlSi10Mg powder.

Machine Parameters	Value	Material Composition			
		Element	wt.%	Element	wt.%
Laser Power	370 W	Al	Balance	Ni	≤0.05
Laser scanning speed	1.3 m/s	Si	9.0–11.0	Pb	≤0.05
Layer thickness	30 μm	Fe	≤0.55	Zn	≤0.10
Beam diameter	0.1 mm	Cu	≤0.05	Sn	≤0.05
Hatch spacing	0.19 mm	Mn	≤0.45	Ti	≤0.15
Powder size	45 ± 10 µm	Mg	0.2–0.45	-	-

## Data Availability

The data generated during the current study are not publicly available due to research copyright arrangements, but are available from the corresponding author on reasonable request.
